# Objective evaluation of chemotherapy-induced peripheral neuropathy using quantitative pain measurement system (Pain Vision^®^), a pilot study

**DOI:** 10.1186/s40780-017-0089-4

**Published:** 2017-07-25

**Authors:** Junya Sato, Megumi Mori, Satoru Nihei, Satoshi Takeuchi, Masahiro Kashiwaba, Kenzo Kudo

**Affiliations:** 1 Department of Hospital Pharmacy, Iwate Medical University, 19-1 Uchimaru, Morioka, Iwate, 020-8505 Japan; 2 Department of Clinical Pharmaceutics, School of Pharmacy, 2-1-1 Nishitokuta, Yahaba, Iwate 028-3694 Japan; 3 Present Address: Department of Pharmacy, Shizuoka Cancer Center, 1007 Shimonagakubo, Nagaizumi-cho, Sunto-gun, Shizuoka 411-8777 Japan; 4 Department of obstetrics and gynecology, School of Medicine, Iwate Medical University, 19-1 Uchimaru, Morioka, Iwate 020-8505 Japan; 5 Department of Breast Surgery, Breastpia Miyazaki Hospital, 2-112-1 Maruyama, Miyazaki, Miyazaki 880-0052 Japan

**Keywords:** Chemotherapy-induced peripheral neuropathy, Quantitative pain measurement system, Pain vision, Objective evaluation, Pain degree

## Abstract

**Background:**

In an evaluation of chemotherapy-induced peripheral neuropathy (CIPN), objectivity may be poor because the evaluation is determined by the patient’s subjective assessment. In such cases, management of neuropathy may be delayed and CIPN symptoms may become severe. In this pilot study, we attempted an objective evaluation of CIPN using a quantitative pain measurement system (Pain Vision^®^).

**Methods:**

The subjects were patients with gynecologic cancer who underwent chemotherapy using taxane and platinum drugs. The grade of the peripheral sensory nerve disorder was based on the Common Terminology Criteria for Adverse Events (CTC-AE) ver. 4.0 and was evaluated before the initiation of therapy and up to six chemotherapy cycles. A symptom scale assessed by the patients using a peripheral neuropathy questionnaire (PNQ) was also evaluated. Simultaneously during these evaluations, graded electric current was applied from the probe to a fingertip and measured both the lowest perceptible current and lowest current perceived as pain by Pain Vision^®^. From these values, the pain degree was calculated from the following formula: (pain perception current value - lowest perceptible current value) ÷ lowest perceptible current value × 100. We compared the pain degrees by Pain Vision^®^ during CIPN development with the value obtained before chemotherapy initiation.

**Results:**

Forty-one patients were enrolled. In the evaluation by a medical professional, 28 (64.3%) patients developed CIPN during 2.5 ± 1.1 chemotherapy cycles (mean ± standard deviation). The pain degree by Pain Vision^®^ at grade 1 and 2 CIPN development according to the evaluation (CTC-AE) was significantly decreased compared to that before chemotherapy initiation (126.0 ± 114.5 vs. 69.8 ± 46.8, *p* = 0.001, and 126.0 ± 114.5 vs. 32.8 ± 32.6, *p* = 0.004). Changes in the pain degree by Pain Vision^®^ were also found during scale B and C, D CIPN development in the patient evaluation (PNQ) (115.9 ± 112.4 vs. 70.6 ± 56.5, *p* = 0.005, and 115.9 ± 112.4 vs. 46.3 ± 42.9, *p* = 0.004). In the 13 patients in whom CIPN did not occur, no significant decrease in the pain degree by Pain Vision^®^ was detected (*p* = 0.764). There was no discontinuation of the measurements because of adverse events such as discomfort from the electric current.

**Conclusion:**

The decrease in the pain degree measured by Pain Vision^®^ was associated with the onset of CIPN symptoms. Particularly, detection of CIPN by Pain Vision^®^ was possible, though most of the CIPN that occurred was low grade or mild symptom. Pain Vision^®^ might become a noninvasive and convenient objective CIPN detection tool to supplement subjective CIPN evaluation.

**Trial registration:**

The study approval number in the institution; H25–140. Registered December 17, 2013.

## Background

Chemotherapy-induced peripheral neuropathy (CIPN) is a refractory side effect developed in a dose-cumulative manner when a taxane, platinum-based anticancer drug, or vinca alkaloid is used. The symptoms of CIPN develop as the glove-and-stocking type of hypesthesia, hyperesthesia, or dysesthesia of the peripheral hands and feet [1]. The region of damage in CIPN spread in size and the degree of symptom progress in a dose- cumulative manner of the responsible anticancer drug. When CIPN becomes severe, it persists in the long term. Furthermore, when CIPN progresses, motor nerve disorders of the hands and feet also develop, causing difficulties with some activities of daily living (ADLs) [2]. ADLs such as walking, writing, changing clothes, and diet are gradually affected by severe CIPN. Therefore, when severe CIPN develops, it interferes with not only ADLs but also quality of life (QOL) in the long term [3].

An antidepressant (tricyclic antidepressant [amitriptyline] [4] or serotonin-noradrenaline reuptake inhibitor [duloxetine] [5]), antiepileptic drug (carbamazepine [6] or gabapentin [7]), or Kampo medicine (Goshajinkigan) [8] is used for prophylaxis and symptomatic relief of CIPN. However, the effects of these drugs are slight or insufficient [9]. Because of the above-mentioned background, early detection and early management are important in CIPN.

Adverse events including CIPN were recorded by a medical professional according to the Common Terminology Criteria for Adverse Events (CTC-AE). The objectivity of a CIPN evaluation performed by a medical professional might be poor in cases where the patient has mild symptomatic expression. In fact, the underestimation of symptoms was pointed out in a comparison of evaluations by medical professionals and patients [10, 11]. The numerical rating scale (NRS) and visual analog scale (VAS) have been used for numerical evaluations of neuropathic pain [12, 13]. The advantages of the NRS and VAS are that they are simple measurements and it is easy to understand changes in a person’s pain. However, pain evaluation by the VAS or NRS was thought to be easily affected by psychological influences including anger and social loneliness [14]. The NRS value may also be influenced by the preference of the number of patients. It may also be difficult for children and the elderly to understand the VAS and NRS. The Functional Assessment of Cancer Therapy-Neurotoxicity (FACT-Ntx) or a patient neurotoxicity questionnaire (PNQ) filled out by the patient was used as another CIPN evaluation method. These evaluation of peripheral neuropathy were determined only by the patient’s subjective assessment. However, patient evaluation with a questionnaire may be inconvenient in clinical practice when there is a large number of questions in the document and when the patients do not understand the questions. Therefore, an objective CIPN evaluation method is required to supplement evaluations by medical professionals and patients.

Pain Vision^®^ (PS-2100 N; Nipro Co., Ltd. Osaka, Japan) is a device for quantitative analyses of perception and painful sensations [15, 16]. The device applies electrical current to the fingertips of subjects from an electrode and quantifies the algetic degree by measuring the lowest perceptible current and the current at which pain is perceived. Pain is transmitted by three kinds of nerve fibers depending on its properties. Aβ fibers transmit the senses of touch and pressure, Aδ fibers transmit mechanical irritation, temperature, and momentary pain, and C fibers transmit burning pain [17]. The Pain Vision^®^ stimulates Aβ and Aδ fibers and evaluates these neurologic functions. The effectiveness of severity determinations has been reported in diabetic neuropathy, post-herpetic neuralgia, and cancer pain using the Pain Vision^®^ [18–20]. These measurements using Pain Vision^®^ are covered under health insurance as an established diagnostic method in Japan. However, an objective evaluation of CIPN using Pain Vision^®^ has not been reported. In this pilot study, we investigated whether CIPN could be evaluated by Pain Vision^®^ in patients undergoing chemotherapy with a taxane and platinum-based anticancer drug.

## Methods

### Study subjects and investigational period

The subjects were patients with gynecologic cancer who underwent chemotherapy with paclitaxel (PTX; 175 mg/m^2^) and a platinum-based chemotherapeutic drug (carboplatin (CBDCA): Area under the blood concentration-time curve (AUC) = 5, cisplatin: 75 mg/m^2^, or nedaplatin: 80 mg/m^2^) with or without bevacizumab (15 mg/kg) every 3 weeks at Iwate Medical University Hospital between July 2014 and November 2015. Among these subjects, we obtained informed consent from incipient patients without diabetes, lymphedema, or rheumatism, and Eastern Cooperative Oncology Group (ECOG) performance status ≥2. We began the investigation with the subjects who agreed to participate in the study. The analysis was performed on subjects who were able to continue more than four cycles of chemotherapy.

### Endpoints

#### CIPN evaluations by a medical professional and the patient

CIPN was evaluated as peripheral sensory neuropathy. The evaluation by a medical professional (primary physician or pharmacist) used a CTC-AE *ver.* 4.0 – Japan Clinical Oncology Group (JCOG). Moreover, an evaluation by the patient was conducted using a PNQ. The PNQ is a method to evaluate neuropathy in five gradations from scale A (no neuropathy) to scale E (severe neuropathy) (Table 1) [11].

**Table 1:** Grading of peripheral sensory neuropathy by the CTCAE ver. 4.0 and PNQ

	Grade 1	Grade 2	Grade 3	Grade 4	Grade 5
CTC-AE ver 4.0	Asymptomatic; loss of deep tendon reflexes or paresthesia	Moderate symptoms; limiting instrumental ADL	Severe symptoms; limiting self care ADL	Life-threatening consequences; urgent intervention indicated	Death
	Scale A	Scale B	Scale C	Scale D	Scale E
PNQ	I have no numbness, pain or tingling in my hand	I have mild tingling, pain or numbness in my hand. This dose not interfere with my ADL.	I have moderate tingling, pain or numbness in my hand. This dose not interfere with my ADL.	I have moderate to severe tingling, pain or numbness in my hand. This interferes with my ADL.	I have severe tingling, pain or numbness in my hand. It completely prevents me from doing most ADL.

#### Evaluation by PainVision^®^

**Fig. 1:**
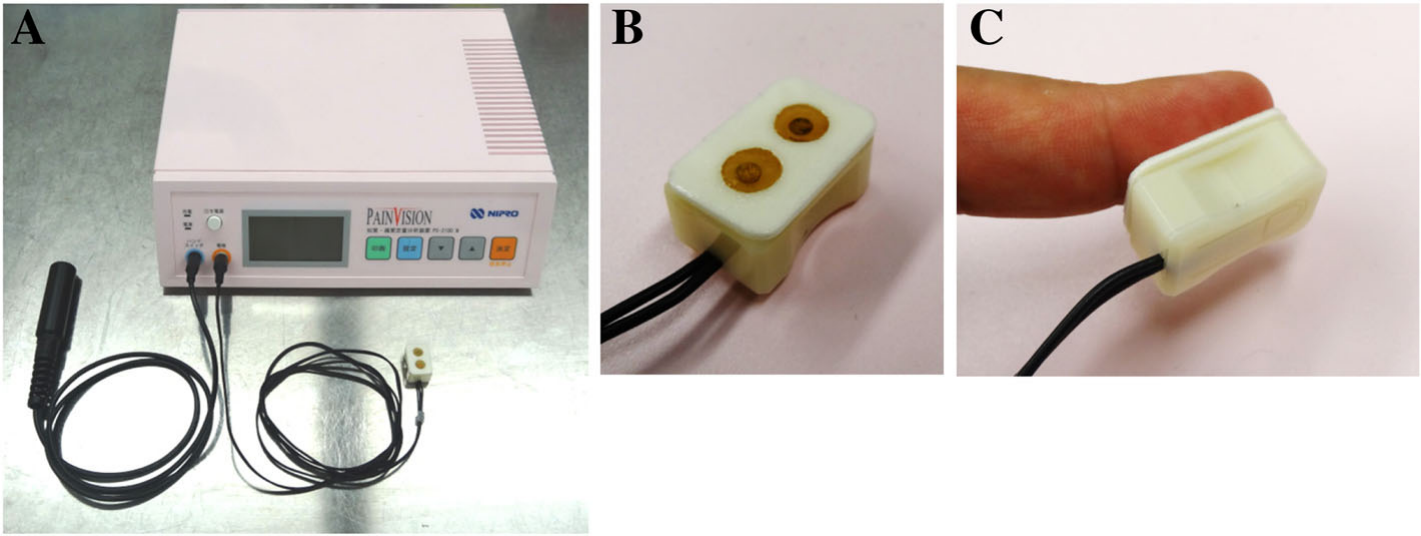
The device for quantitative analyses of perception and painful sensations and its probe. Indicated the device for quantitative analyses of perception and painful sensations, (**a**) The PainVision^®^ PS-2100 N main device, (**b**) fingertip probe, and (**c**) attached fingertip probe. When the subject sensed the electric current for the first time or sensed the electric current as pain, the subject pushed a hand switch. The current at these point were determined as the lowest perceptible current and the pain perception current, respectively

The evaluation of CIPN by Pain Vision^®^ was performed by affixing a probe (EL-PATCH; Nipro Co., Ltd.) to a fingertip (Fig. 1). For the stimulation conditions, the voltage and current limits were set to 200 V and 256 μA, respectively. The current applied by the probe increased over time. The duration in which the electric current achieved its maximal limit was set to 30 s. When the subject sensed the electric current for the first time, the subject pushed a hand switch. The current at this point was determined as the lowest perceptible current. Then, when the subject first sensed the electric current as pain, the current at this point was determined as the pain perception current. The measurement was carried out twice and used the mean numerical value of both electric current values. According to a previous report [16], the pain degree was calculated using the following formula: (pain perception current - lowest perceptible current) ÷ lowest perceptible current × 100. Every measurement by Pain Vision^®^, we determined an adverse event by the measurement.

### Evaluation timing and analysis

Each evaluation was carried out before the initiation of each cycle of chemotherapy and for to up to six cycles. The main analysis was performed by comparing the pain degree by Pain Vision^®^ after symptom development to that at chemotherapy initiation by a medical professional and by patient evaluation among patients in whom CIPN occurred. Moreover, changes in the pain degree by Pain Vision^®^ among patients in whom CIPN did not occur were evaluated sequentially.

### Statistical analysis

Changes in the pain degree by Pain Vision^®^ between patients before chemotherapy initiation and after CIPN symptom development evaluated by CTC-AE or PNQ were examined by one-way analysis of variance (ANOVA), and multiple comparison by Fisher’s least significant difference (Fisher’s LSD) test was performed before chemotherapy initiation as control. Changes in the pain degree by Pain Vision^®^ in patients in whom CIPN did not occur was also examined by one-way ANOVA, and then differences in the pain degree by Pain Vision^®^ between chemotherapy initiation and after each cycle were tested using multiple comparison tests (Dunnett test). Both tests were judged as significant at a 5% level for hazard ratios. The statistics software used was Excel statistics 2012 (Social Survey Research Information Co Ltd., Tokyo, Japan).

### Ethical permission

This study conducted with the permission of the Iwate Medical University School of Medicine Ethical Review Board (H25–140).

## Results

### Patient background

**Fig. 2:**
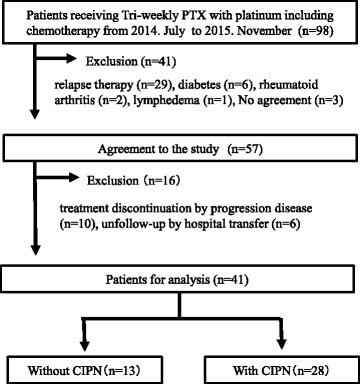
The CONSORT diagram of the study patients. Indicated the CONSORT diagram of the study patients. Fifty-seven patients agreed to the participation in the study period. Forty-one patients who were able to continue chemotherapy were analyzed

A CONSORT diagram for this study was shown in Fig. 2. During the study period, 98 patients underwent chemotherapy with PTX and a platinum-based drug every 3 weeks. Of these, 41 patients undergoing relapse therapy (*n* = 29) and those with diabetes (*n* = 6), rheumatoid arthritis (*n* = 2), lymphedema (*n* = 1), and those who did not agree to participate (*n* = 3) were excluded from the study. The CIPN evaluations of the 57 patients who agreed to participate in the study were performed continuously. The analysis was performed on 41 patients after excluding 10 patients who discontinued treatment because of disease progression and 6 patients lost to follow-up because of a hospital transfer. The patients’ backgrounds for these analyses were presented in Table 2.

**Table 2:** Backgrounds of the patients for analysis

		All patient (*n* = 41)	With CIPN (*n* = 28)	Without CIPN (*n* = 13)
Age		55.8 ± 11.0	57.0 ± 10.7	53.2 ± 11.6
Cancer type	Ovarian	22	16	6
	Cervical	11	5	6
	Endometrial	8	7	1
Regimen	TC ± BV	34	27	7
	TP	5	1	4
	PTX + NED	2	0	2
Cycle		5.9 ± 0.4	5.9 ± 0.3	5.7 ± 0.7
Body-surface area (m^2^)		1.484 ± 0.126	1.472 ± 0.121	1.509 ± 0.137
Cumulative PTX dose (mg/m^2^)		1000.6 ± 101.3	1028.5 ± 38.6	940.8 ± 158.6
Cumulative Platinum dose (mg/m^2^)	CBDCA	2328.9 ± 305.5	2340.7 ± 294.5	2283.6 ± 366.7
	CDDP	514.8 ± 112.4	296	331.8 ± 35.1
	NED	604.0 ± 5.7	-	400

Values are indicated as means ±standard deviations

*PTX* paclitaxel, *CBDCA* carboplatin, *CDDP* cisplatin, *NED* nedaplatin, *TC ± BV* bevacizumab (15 mg/kg) ± paclitaxel (175 mg/m^2^) + carboplatin (AUC = 6), *TP* paclitaxel (175 mg/m^2^) + cisplatin (75 mg/m^2^), *PTX-NED* paclitaxel (175 mg/m^2^) + nedaplatin (80 mg/m^2^)

### CIPN symptoms and the pain degree by pain vision^®^

**Fig. 3:**
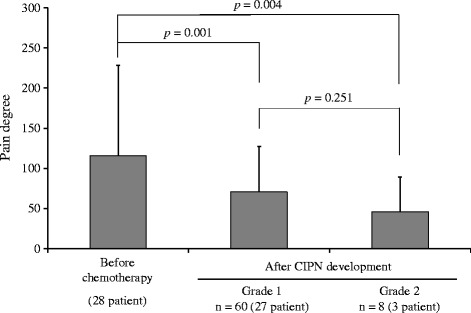
Changes in the pain degree by Pain Vision^®^ at CIPN development as evaluated by a medical professional (CTCAE ver. 4.0). Indicated changes in the pain degree by Pain Vision^®^ at CIPN development as evaluated by a medical professional (CTCAE ver. 4.0). The bar indicates the pain degree by Pain Vision^®^ as means ± standard deviations before chemotherapy and after CIPN development (grades 1 and 2). A comparison of both values was performed with Fisher’s LSD test

**Fig. 4:**
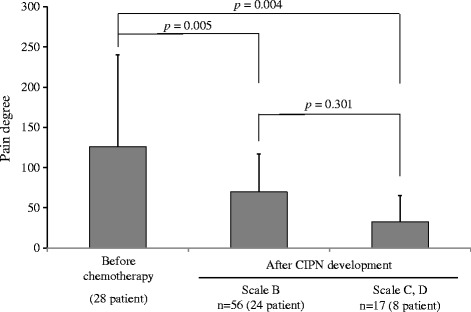
Changes in the pain degree by Pain Vision^®^ at CIPN development as evaluated by the patient (PNQ). Indicated changes in the pain degree by Pain Vision^®^ at CIPN development as evaluated by the patient (PNQ). The bar indicates the pain degree by Pain Vision^®^ as means ± standard deviations before chemotherapy and after CIPN development (Scales B and C, D). A comparison of both values was performed with Fisher’s LSD test

CIPN occurred in 28 (68.3%) patients from 2.5 ± 1.1 (mean ± standard deviation) chemotherapy cycles in the evaluation by a medical professional (CTC-AE). In these patients, severe CIPN (CTC-AE grade ≥ 3 or PNQ scale E) did not occur. During the 68 cycles in which CIPN was detected in these 28 patients in the evaluation by a medical professional (CTC-AE). Grade 1 and 2 of CIPN finally occurred in 60 cycles (27 patients) and 8 cycles (3 patients), respectively. Among 3 patients who develop grade 2, 1 patient was grade 2 since onset of symptom. Others 2 patients developed grade 2 after they experienced grade 1. The pain degree by Pain Vision^®^ at symptom development (grades 1 and 2) significantly decreased compared with that before chemotherapy initiation (*p* = 0.024 by ANOVA and grade 1 [*n* = 60 (27 patients)]: 126.0 ± 114.5 vs. 69.8 ± 46.8, *p* = 0.001; grade 2 [*n* = 8 (3 patients)]: 126.0 ± 114.5 vs. 32.8 ± 32.6, *p* = 0.004). However, the significant difference of the pain degree between grade 1 and 2 was not observed (*p* = 0.251, by Fisher’s LSD test) (Fig. 3). Similarly, during the 73 cycles in which CIPN was detected in 28 patients by the patient evaluation (PNQ). Scale B and C, D of CIPN finally occurred in 56 cycles (24 patients) and 17 cycles (8 patients), respectively. Among 8 patients who develop scale C, D, 4 patients were scale C, D since onset of symptom. Others 4 patients developed scale C, D after they experienced scale B. The pain degree by Pain Vision^®^ at symptom development (scales B and C, D) significantly decreased compared with that before chemotherapy initiation (*p* = 0.024 by ANOVA and scales B [*n* = 56 (24 patients)]: 115.9 ± 112.4 vs. 70.6 ± 56.5, *p* = 0.005; scales C, D [*n* = 17 (8 patients)]: 115.9 ± 112.4 vs. 46.3 ± 42.9, *p* = 0.004). However, the significant difference of the pain degree between scale B and C, D was not observed (*p* = 0.301, by Fisher’s LSD test) (Fig. 4). In the 13 patients in whom CIPN did not occur, the pain degrees by Pain Vision^®^ during cycles 1–6 were 136.0 ± 174.5 (13 patient), 136.6 ± 167.4 (13 patient), 78.1 ± 113.0 (12 patient), 115.0 ± 127.8 (12 patient), 289.0 ± 521.8 (12 patient), and 407.6 ± 793.3 (8 patient), respectively. No significant decrease in the pain degree by Pain Vision^®^ was detected as compared with cycle 1(before the chemotherapy initiation) (*p* = 0.325 by ANOVA).

### Adverse event

The measurement by PainVision^®^ did not cause discomfort from the electric current flowing to the fingertip, and there was no discontinuation of the measurement by patient request.

## Discussion

It was reported that evaluations by Pain Vision^®^ were rarely affected by psychogenic factors [21]. Fujii et al. evaluated the effect of lidocaine on neuropathic pain using the VAS and Pain Vision^®^. They considered that an evaluation by Pain Vision^®^ was able to remove the influence of a placebo effect [19]. Pain Vision^®^ was effective for an objective evaluation of neuropathic pain. However, an investigation of CIPN has not been previously reported. To our knowledge, this study was the first pilot study to investigate the potential of Pain Vision^®^ for the objective evaluation of CIPN.

In the present results, the decrease in the pain degree measured by Pain Vision^®^ was associated with the onset of CIPN symptoms. Though most of the CIPN that occurred featured comparatively mild symptoms (grade 1 by the CTC-AE and scale B by the PNQ), a significant decrease in the pain degree was detected by Pain Vision^®^. In this study using Pain Vision^®^, we determined the lowest perceptible current and the current at which pain was perceived, and calculated the pain degree. Correction by the lowest perceptible current reduces the variation in the measurement result caused by measurement conditions such as the positions of the electrode and subcutaneous nerves and individual differences in how patients feel pain [17]. It seemed that the decrease in the pain degree by Pain Vision^®^ at CIPN development was caused by an increase in the lowest perceptible current from hypesthesia, a decrease in the current perceived to cause pain from a decrease in the pain threshold, or both of these changes. It was thought that this neuropathic etiology was primarily due to PTX. PTX causes CIPN according to its cumulative dose. In patients with metastatic breast cancer, the mean cumulative PTX dose at which CIPN develops at grade 2 or greater was reported as 715 mg/m^2^ [22]. Because the mean cumulative dose of PTX was 1000 mg/m^2^ or more, CIPN induced by PTX seemed certain to occur. Moreover, the percentages of CIPN of all grades in a previous report were 15.4% and 46.9% in a comparison of pegylated liposomal doxorubicin + CBDCA and PTX + CBDCA therapy for the first occurrence of ovarian cancer, respectively [23]. From the considerable difference in these CIPN incidences, most CIPN etiologies were thought to be induced by PTX. The peripheral neuropathy induced by PTX is due to axonal denaturation with inhibition of peripheral axonal microtubule polymerization [24]. It is thereby felt as hypoesthesia or dysesthesia such as the numbness of the dominant nerve field. Pain Vision^®^ stimulates Aβ fibers responsible for sensing touch and pressure efficiently and quantifies their sensitivity. Therefore, it was thought that Pain Vision^®^ sensed dysesthesia caused by PTX as a decrease in the pain degree as well.

The limitations of this study and details that we should consider in future were as follows. Primarily, it should be considered whether the detection of CIPN by Pain Vision^®^ could be replicated with other anticancer drugs (oxaliplatin, a vinca alkaloid, etc.) besides PTX. Secondly, 10 patients in whom CIPN did not occur and five patients who had CIPN in this study were using a drug that may relieve peripheral neuropathy (Goshajinkigan: *n* = 8, mecobalamin: *n* = 3, oxycodone: *n* = 3, duloxetine: *n* = 1, pregabalin: *n* = 1, tramadol: *n* = 1). The use of these drugs might influence the perception of current in the Pain Vision^®^ evaluation and CIPN symptoms. An investigation is required in the future regarding the change in the pain degree by Pain Vision^®^ before and after the use of drugs providing CIPN relief and when symptoms were relieved by these drugs. Furthermore, an investigation is required regarding the magnitude of the change in the pain degree by Pain Vision^®^ at which we should suspect CIPN development and whether there is a cutoff value for the change in the pain degree by Pain Vision^®^. The decrease in the pain degree appeared to correlate with the progression of symptoms. However, some patients had no correlation between threshold level by pain vision^®^ and medical interview (CTCAE and PNQ). While medical professional evaluation (CTC-AE) showed CIPN in 68 cases (28 patients), the pain degree did not decrease in 21 cases (13 patients). Similar observation was present in 23 cases (11 patients) in patient evaluation (PNQ). Psychogenic effect was considered as the explanation. For example, even if CIPN does not occur physiologically, patients might notice a symptom. A sense of fear for the electric current or habituation might also affect a reaction time to push the switch. As other reasons, dry condition of the finger, adhesiveness of the electrode to a finger-tip might affect the threshold. There were also no significant differences in the pain degree between patients with CTC-AE grade 1 and grade 2. Similarly, even PNQ scale was similar. Therefore, it was believed that there were only 3 and 8 patients with CTC-AE grade 2 and PNQ scale, respectively. Future studies with larger sample size are warranted.

## Conclusion

The possibility that Pain Vision^®^ might become a noninvasive, simple, and easy-to-perform objective evaluation procedure to supplement existing CIPN evaluations was suggested by the present results.

## Abbreviations

ADLs: Activities of daily living
ANOVA: One-way analysis of variance
CBDCA: Carboplatin
CIPN: Chemotherapy-induced peripheral neuropathy
CTC-AE: Common terminology criteria for adverse events
ECOG: Eastern cooperative oncology group
Fisher’s LSD: Fisher’s least significant difference
JCOG: Japan clinical oncology group
NRS: Numerical rating scale
PNQ: Patient neurotoxicity questionnaire
PTX: Paclitaxel
QOL: quality of life
VAS: Visual analog scale
